# Laparoscopic hepatic flexure mobilisation

**DOI:** 10.1308/003588412X13373405385214c

**Published:** 2012-07

**Authors:** ER MacDonald, AA Renwick, RG Molloy

**Affiliations:** ^1^Aberdeen Royal Infirmary,UK; ^2^Royal Alexandra Hospital, Paisley,UK; ^3^Gartnavel General Hospital, Glasgow,UK

**Figure 1:**
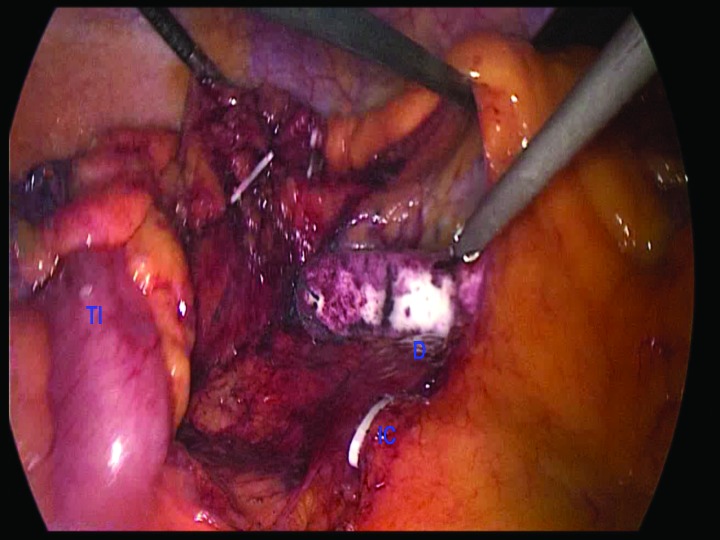
Placement of swab in front of duodenum

**Figure 2:**
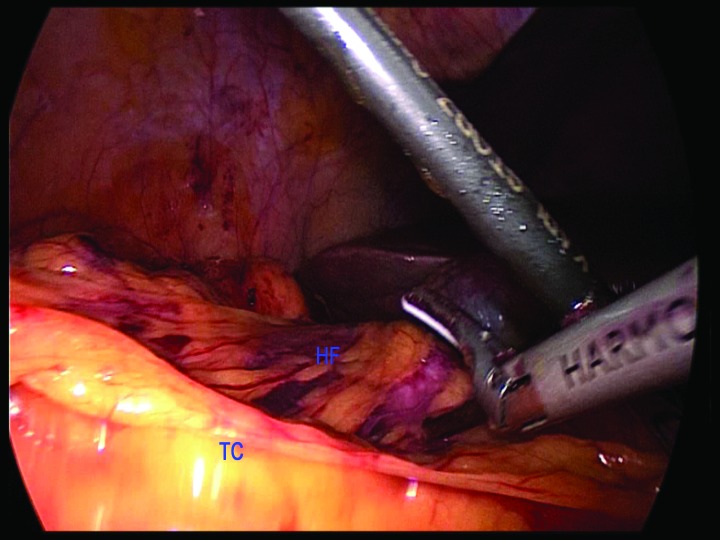
Identification of swab from above at hepatic flexure

We describe a simple method to help identify the correct plane for hepatic flexure mobilisation while simultaneously protecting the duodenum during a laparoscopic right hemicolectomy. The placement of a swab or nasal pack on top of the duodenum after medial to lateral dissection below the ileocolic pedicle and right mesocolon (Fig 1) allows clear identification of the hepatic flexure from above. This can then be divided safely with the Harmonic® scalpel (Ethicon Endo-Surgery, Cincinnati, OH, US) protecting the duodenum from thermal injury (Fig 2).

TI = terminal ileum; D = duodenum; IC = ileocolic pedicle

HF = hepatic flexure; TC = transverse colon

